# Multiple uncontrolled conditions and blood pressure medication intensification: an observational study

**DOI:** 10.1186/1748-5908-5-55

**Published:** 2010-07-19

**Authors:** Amanda H Salanitro, Ellen Funkhouser, Bonita S Agee, Jeroan J Allison, Jewell H Halanych, Thomas K Houston, Mark S Litaker, Deborah A Levine, Monika M Safford

**Affiliations:** 1 VA National Quality Scholars Program, Department of Veterans Affairs Medical Center, Birmingham, AL, USA; 2 Center for Surgical, Medical Acute care Research and Transitions (C-SMART), Department of Veterans Affairs Medical Center, Birmingham, AL, USA; 3 Division of General Internal Medicine, University of Alabama at Birmingham, Birmingham, AL, USA; 4 Preventive Medicine, University of Alabama at Birmingham, Birmingham, AL, USA; 5 Department of Quantitative Health Sciences and Medicine, University of Massachusetts Medical School, Worcester, MA, USA; 6 Center for Health Quality, Outcomes & Economic Research (CHQOER), Department of Veterans Affairs Medical Center, Bedford, MA, USA; 7 Department of General Dental Sciences, University of Alabama at Birmingham, Birmingham, AL, USA; 8 Ann Arbor VA Medical Center/University of Michigan Health System Patient Safety Enhancement Program, Department of Veterans Affairs Medical Center, Ann Arbor, MI, USA; 9 Division of General Medicine, University of Michigan Medical School, Ann Arbor, MI, USA

## Abstract

**Background:**

Multiple uncontrolled medical conditions may act as competing demands for clinical decision making. We hypothesized that multiple uncontrolled cardiovascular risk factors would decrease blood pressure (BP) medication intensification among uncontrolled hypertensive patients.

**Methods:**

We observed 946 encounters at two VA primary care clinics from May through August 2006. After each encounter, clinicians recorded BP medication intensification (BP medication was added or titrated). Demographic, clinical, and laboratory information were collected from the medical record. We examined BP medication intensification by presence and control of diabetes and/or hyperlipidemia. 'Uncontrolled' was defined as hemoglobin A1c ≥ for diabetes, BP ≥ 140/90 mmHg (≥ 130/80 mmHg if diabetes present) for hypertension, and low density lipoprotein cholesterol (LDL-c) ≥ 130 mg/dl (≥ 100 mg/dl if diabetes present) for hyperlipidemia. Hierarchical regression models accounted for patient clustering and adjusted medication intensification for age, systolic BP, and number of medications.

**Results:**

Among 387 patients with uncontrolled hypertension, 51.4% had diabetes (25.3% were uncontrolled) and 73.4% had hyperlipidemia (22.7% were uncontrolled). The BP medication intensification rate was 34.9% overall, but higher in individuals with uncontrolled diabetes and uncontrolled hyperlipidemia: 52.8% overall and 70.6% if systolic BP ≥ 10 mmHg above goal. Intensification rates were lowest if diabetes or hyperlipidemia were controlled, lower than if diabetes or hyperlipidemia were not present. Multivariable adjustment yielded similar results.

**Conclusions:**

The presence of uncontrolled diabetes and hyperlipidemia was associated with more guideline-concordant hypertension care, particularly if BP was far from goal. Efforts to understand and improve BP medication intensification in patients with controlled diabetes and/or hyperlipidemia are warranted.

## Background

As the US population ages, increasing numbers of individuals have multiple medical conditions [1,2]. As a result, primary care clinicians are increasingly seeing patients with multiple conditions, such as diabetes and/or hyperlipidemia, that contribute to increased risk of cardiovascular disease (CVD). Although the two conditions frequently occur in the same patient, little information is available regarding how to prioritize disease management and implement guideline-concordant care for complex patients with multiple uncontrolled conditions [1-3]. In practice, clinicians manage uncontrolled conditions inconsistently at every clinical encounter [4-7], and rates of simultaneous control of all co-existing conditions are suboptimal [8].

The phenomenon of patients presenting for care with uncontrolled conditions or risk factors and the clinician not adjusting the medication regimen has been called 'clinical inertia' [9,10]. In primary care practice, there are situations in which clinicians may choose not to follow disease-specific guidelines or adhere to quality measures based on clinically appropriate reasons. While reports of clinical inertia are accumulating, the reasons why it occurs remain limited [11]. In addition, it is unknown whether the presence of multiple uncontrolled conditions contributes to clinical inertia.

To examine the role of multiple uncontrolled conditions in clinical inertia, we studied the association of blood pressure (BP) intensification and the presence and control of two other CVD risk factors, diabetes and hyperlipidemia, in patients with uncontrolled hypertension. We hypothesized that additional uncontrolled CVD risk factors would act as competing demands, decreasing the likelihood that clinicians would adjust BP medications when the patient's BP was above goal.

## Methods

### Setting and design

This study was conducted at two VA Medical Center primary care clinics in 2006. Of the 15 clinicians practicing at the clinics, 13 chose to participate. We observed all consecutive patient visits made to each clinician over the observation period (two to nine weeks during May to August 2006). Patient visits occurred on six to sixteen half-day sessions of clinic and included 37 to 151 patient visits, depending on the clinician.

### Data sources

At each visit, research assistants recorded the patient's BP as assessed by the nursing assistant or nurse, and the value and date of the most recent hemoglobin A1c and low density lipoprotein cholesterol (LDL-c) levels as recorded in the medical record. A data collection tool including this information was provided to the clinician prior to seeing each patient. At the conclusion of the visit, the clinicians recorded whether BP medications were intensified. The BP, diabetes, and lipid medications at the time of the visit were also recorded. The Birmingham VA Medical Center's Institutional Review Board approved the study protocol.

### Study sample

We observed 946 consecutive patients and include in this analysis the 387 patients who presented with uncontrolled BP. Patients who were seen more than once only had their first visit analyzed. Uncontrolled BP was defined as ≥ 140/90 mmHg, or ≥ 130/80 if the patient had diabetes. Diabetes was defined according to Miller's definition: more than one ICD-9-CM code for diabetes (250.xx) within two years prior to the observed visit, or a prescription for any diabetes medication in the current year [12]. Hyperlipidemia was defined as being on a lipid-lowering medication within two years prior to the observed visit, or an LDL-c ≥ 130 mg/dL at the visit, or ≥ 100 mg/dL if the patient had diabetes.

### Dependent variable: BP medications intensified

We defined BP medication as intensified if the clinician indicated, on the data collection form, addition of a new medication or increase in the dose of an existing medication. Clinicians indicated whether they intensified medications in all but 17 patients with uncontrolled high BP. The 17 patients without indication of intensification were classified as not intensified.

### Main exposure: uncontrolled CVD risk factors

The main exposures of interest were uncontrolled diabetes and uncontrolled hyperlipidemia. Uncontrolled diabetes was defined as most recent recorded A1c ≥ 7%. Uncontrolled hyperlipidemia was defined as most recent recorded LDL-c ≥ 130 mg/dL, or ≥ 100 mg/dL if the patient had diabetes.

Because patients were consecutive primary care patients, not all had diabetes or hyperlipidemia. Therefore, for each patient we indicated which of the following four mutually exclusive clinical scenarios was present: uncontrolled hypertension and no diagnosed diabetes or hyperlipidemia; controlled diabetes and/or hyperlipidemia; either uncontrolled diabetes or uncontrolled hyperlipidemia; or both uncontrolled diabetes and uncontrolled hyperlipidemia. Because our primary interest was the effect of multiple uncontrolled conditions, patients with known diabetes or hyperlipidemia but no A1c or LDL-C in the record were categorized as having the respective controlled condition. While other clinical scenarios are possible, they occurred too rarely in our sample for separate categorization.

### Other independent variables: patient characteristics

Additional variables included age, gender, systolic and diastolic BP values, and the total number of BP, diabetes, and lipid medications prescribed in the 90 days prior to the observed visit. Because so many patients who met the uncontrolled BP criterion were very near the threshold, we also constructed a variable reflecting mmHg of systolic BP above goal.

### Analysis

Patient age, gender, total number of medications for hypertension, diabetes, hyperlipidemia, BP level, and mmHg above goal were examined across the four clinical scenarios using Chi-square tests, analysis of variance, and Kruskal-Wallis tests where appropriate. In addition, we examined the proportions of patients whose BP medications were intensified for each patient characteristic, for each risk factor separately, and for combinations of elevated risk factors. Hierarchical logistic regression models were constructed to identify independent associations with BP medication intensification, adjusting for clustering of patients within clinician and for patient factors. The main exposure of interest was a categorical variable representing the four clinical scenarios defined above. The referent category was the scenario where the patient had uncontrolled BP and controlled diabetes and/or hyperlipidemia. All variables were retained in the multivariable model except patient gender, due to small numbers of women. We estimated adjusted odds ratios (OR) and predicted probabilities, each with 95% confidence intervals (CI), from these models. The predicted probabilities of BP medication intensification for each of the four clinical scenarios were calculated from these models using the sample mean age, systolic BP, and sample median number of total medications for hypertension, diabetes, and hyperlipidemia.

Preliminary analyses indicated that intensification rates were strongly associated with systolic BP level, with lower rates for those with lower systolic BP. To examine the robustness of the effect of multiple uncontrolled conditions at even low elevations of systolic BP, we stratified the sample on the median mmHg above the goal, which was 10 mmHg. Therefore, the near-goal sample included individuals with systolic BP of 130 to 139 mmHg if they had diabetes, and 140 to 149 mmHg if they did not. We also examined the effect of restricting the analysis to only patients known to have hyperlipidemia and/or diabetes, again stratifying on the median mmHg above goal (within 10 mmHg). Finally, we conducted a sensitivity analysis that excluded patients without A1c or LDL-c assessed from the analysis, rather than grouping them as controlled.

## Results

The 387 patients had mean age of 63 ± 13 years (range: 25 to 90), 3.6% were female, 51.4% had diabetes and 77.3% had hyperlipidemia. The mean systolic BP was 146 ± 15 mmHg (range: 113 to 208), and 52.5% had systolic BP level within 10 mmHg of their goal. The median number of medications prescribed for hypertension, diabetes, and hyperlipidemia at the time of the visit was 2 (range: 0 to 9). The proportion presenting with uncontrolled diabetes or uncontrolled lipids was similar (25.3% versus 22.7%, respectively). As expected, patient characteristics differed across the four clinical scenario groups (Table 1). Patients with neither diabetes nor hyperlipidemia were younger and had lower systolic BP, and patients with uncontrolled conditions were on more medications.

**Table 1:** Characteristics of 387 patients presenting with uncontrolled blood pressure, overall and by clinical scenario

			Clinical Scenario: Uncontrolled BP AND								
Patient Factors	All		No diagnosed diabetes or HLD† (N = 76)		Controlled diabetes and/or HLD (N = 157)		Either uncontrolled diabetes or HLD (N = 122)		Both uncontrolled diabetes and HLD (N = 32)		P
	N	%	N	%	N	%	N	%	N	%	
Age (years)											< 0.001
< 55	92	23.8%	32	42.1%	22	14.0%	31	25.4%	7	21.9%	
55 to 64	136	35.1%	15	19.7%	56	35.7%	50	41.0%	15	46.9%	
65+	159	41.1%	29	38.2%	79	50.3%	41	33.6%	10	31.2%	
Gender											0.80
Male	373	96.4%	74	97.4%	152	96.8%	117	95.9%	30	93.7%	
Female	14	3.6%	2	2.6%	5	3.2%	5	4.1%	2	6.3%	
Systolic BP (mmHg)											< 0.001
< 140	122	31.5%	12	15.8%	39	24.8%	55	45.1%	16	50.0%	
140 to 159	196	50.6%	52	68.4%	89	56.7%	44	36.1%	11	34.4%	
160+	69	17.8%	12	15.8%	29	18.5%	23	18.8%	5	15.6%	
Minimally (0 to 9 mmHg) above goal											0.13
Yes	203	52.5%	49	64.5%	78	49.7%	61	50.0%	15	46.9%	
No	184	47.5%	27	35.5%	79	50.3%	61	50.0%	17	53.1%	
Number of medications*											< 0.01
0	122	31.5%	44	36.1%	51	41.8%	23	18.8%	4	3.3%	
1 to 2	114	29.5%	22	19.3%	48	42.1%	37	32.5%	7	6.1%	
3 to 4	97	25.1%	9	9.3%	35	36.1%	39	40.2%	14	14.4%	
5+	54	13.9%	1	1.8%	23	42.6%	23	42.6%	7	13.0%	
Diabetes											< 0.001
Uncontrolled	98	25.3%	0	0.0%	0	0.0%	66	54.1%	32	100.0%	
Controlled**	101	26.1%	0	0.0%	69	43.9%	32	26.2%	0	0.0%	
Not present	188	48.6%	76	100.0%	88	56.1%	24	19.7%	0	0.0%	
HLD†											< 0.001
Uncontrolled	88	22.7%	0	0.0%	0	0.0%	56	45.9%	32	100.0%	
Controlled**	196	50.7%	0	0.0%	143	91.1%	53	43.4%	0	0.0%	
Not present	103	26.6%	76	100.0%	14	8.9%	13	10.7%	0	0.0%	

*Medications for hypertension, diabetes, and/or hyperlipidemia prescribed in the 90 days prior to the index visit; **Includes missing laboratory data: 23 A1c data, 41 lipids; †HLD = Hyperlipidemia

Overall, 34.9% (135/387) of patients had their BP medications intensified at the visit. In bivariate analysis (Table 2), age and gender had non-significant associations with intensification. Both the absolute level of systolic BP and the number of mmHg above goal were strongly and linearly associated with intensification, which were the only significant bivariate associations with BP medication intensification observed. Intensification rates trended downward (non-significantly) with increasing numbers of medications currently prescribed. When we restricted the number of medications to include antihypertensives only, we found this effect was primarily attributable to the antihypertensive medications (data not shown). Patients with controlled diabetes were intensified at a lower rate (29.7%) than those with uncontrolled diabetes (36.7%) or those who did not have diabetes (36.7%). Intensification rates were similar for patients with uncontrolled (32.0%) and controlled hyperlipidemia (33.2%), both at a lower rate than patients without hyperlipidemia (42.1%). We also found similar intensification rates for patients with diabetes and hyperlipidemia separately (data not shown). There was no difference in BP medication intensification for patients with uncontrolled diabetes versus uncontrolled hyperlipidemia.

**Table 2:** Bivariate associations with blood pressure medication intensification among 387 patients presenting with uncontrolled blood pressure

	BP medication intensification rates		Odds ratio	95% Confidence Interval		
				
Patient Factors	# intensified/total #	%		lower limit	upper limit	P
Age (years)						
< 55	28/92	30.4	0.61	0.35	1.06	0.29
55 to 64	57/136	41.9	Referent			
≥ 65	50/159	31.5	0.64	0.39	1.03	0.36
Sex						
Male	133/373	35.7	Referent			
Female	2/14	14.3	0.3	0.07	1.36	0.12
Systolic BP (mmHg) (mean)						
< 140	21/122	17.2	Referent			
140 to 159	72/196	36.7	2.79	1.61	4.85	0.93
≥ 160	42/69	60.9	7.48	3.81	14.68	< 0.001
Systolic BP < 10 mmHg above goal						
Yes	39/203	19.2	Referent			
No	96/184	52.2	4.59	2.92	7.22	< 0.01
Number of medications*						
0	39/122	31.5	0.68	0.35	1.33	0.35
1 to 2	40/114	29.5	Referent			
3 to 4	34/97	25.1	0.79	0.40	1.53	0.89
5+	22/54	13.9	0.79	0.40	1.56	0.89
Diabetes						
Present-Uncontrolled	36/98	36.7	1.37	0.76	2.49	0.52
Present-Controlled**	30/101	29.7	Referent			
Not present	69/188	36.7	1.37	0.82	2.30	0.46
Hyperlipidemia						
Present-Uncontrolled	33/103	32.0	1.46	0.87	2.45	0.11
Present-Controlled**	65/196	33.2	Referent			
Not present	37/88	42.1	0.95	0.57	1.58	0.33
Clinical scenario: Uncontrolled BP **AND**						
No diagnosed diabetes or HLD†	30/76	39.5	1.53	0.86	2.71	0.72
Controlled diabetes and/or HLD	47/157	29.9	Referent			
Either uncontrolled diabetes or HLD	43/122	35.3	1.27	0.77	2.11	0.56
Both uncontrolled diabetes and HLD	15/32	46.9	2.07	0.95	4.48	0.18

*Medications for hypertension, diabetes, and/or hyperlipidemia prescribed in the 90 days prior to the index visit; **Includes missing laboratory data: 23 hemoglobin A1c levels and 41 lipid levels; †HLD = Hyperlipidemia

We observed a U-shaped relationship between the clinical scenarios and intensification in bivariate analyses, although the odds ratios were not statistically significant (Table 2). That is, the lowest intensification rates were among those with controlled diabetes or hyperlipidemia, compared with those without either condition or with uncontrolled diabetes or hyperlipidemia. BP medication intensification rates increased as the number of total uncontrolled conditions rose; however, patients without either condition were intensified at a higher rate than those patients with either controlled diabetes or hyperlipidemia. These associations strengthened when adjusted for: age; systolic BP level; number of hypertension, diabetes, and hyperlipidemia medications; and clustering (Table 3). The U-shaped relationship was maintained for the group with systolic BP elevation < 10 mmHg and ≥ 10 mm Hg, although it was statistically significant only in the latter group.

**Table 3:** Adjusted odds ratios of blood pressure medication intensification for patients with uncontrolled blood pressure

		95% Confidence Interval		
Clinical scenario: Uncontrolled BP AND	Odds ratio*	lower limit	upper limit	P
**ALL (N = 387)**				

No diagnosed diabetes or hyperlipidemia	1.86	1.04	3.33	0.04
Controlled diabetes and/or hyperlipidemia	Referent			
Either uncontrolled diabetes or hyperlipidemia	1.54	0.97	2.43	0.07
Both uncontrolled diabetes and hyperlipidemia	2.91	1.48	5.72	< 0.01
				
**Systolic BP < 10 mmHg above goal (N = 203)**				

No diagnosed diabetes or hyperlipidemia	1.17	0.41	3.34	0.77
Controlled diabetes and/or hyperlipidemia	Referent			
Either uncontrolled diabetes or hyperlipidemia	0.92	0.43	1.94	0.82
Both uncontrolled diabetes and hyperlipidemia	1.69	0.26	11.07	0.58
				
**Systolic BP ≥ 10 mm Hg above goal (N = 184)**				

No diagnosed diabetes or hyperlipidemia	2.58	1.17	5.67	0.02
Controlled diabetes and/or hyperlipidemia	Referent			
Either uncontrolled diabetes or hyperlipidemia	1.93	0.95	3.93	0.07
Both uncontrolled diabetes and hyperlipidemia	4.51	1.46	13.94	0.01

*Adjusted for age, systolic blood pressure, number of medications and clustering of patients by provider

**Figure 1:**
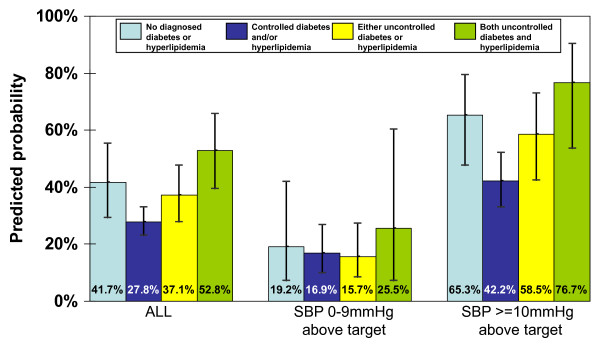
**Predicted probabilities of blood pressure medication intensification by clinical scenario among uncontrolled hypertensive patients**. Error bars represent 95% confidence intervals.

Those with uncontrolled BP and either controlled diabetes or hyperlipidemia (but not both) had the lowest rates of intensification, and those with all three uncontrolled conditions had the highest. This pattern was only statistically significant among the group with the highest BP. Intensification rates and the probability of intensifying were markedly higher for the group further from BP goal compared to those with minimally uncontrolled BP (Table 3; Figure 1). Restricting the analysis to patients with diabetes and hyperlipidemia revealed similar increasing odds of BP medication intensification as the number of uncontrolled conditions rose, although the odds were only significant in the group with all three uncontrolled conditions and systolic BP at least 10 mm Hg above goal (odds ratio 4.55, 95% confidence interval 1.43 to 14.44, p = 0.01). Results were similar in the sensitivity analysis in which we excluded patients with missing A1c and LDL-c values, rather than grouping them as controlled (data not shown).

## Discussion

In this study, patients with multiple uncontrolled CVD risk factors were more likely to have BP medication intensified, contrary to our expectations. Our hypothesis that multiple uncontrolled CVD risk factors, specifically diabetes and hyperlipidemia, would act as competing demands and thereby distract clinicians from treating elevated BP was not supported. Higher BP medication intensification rates in patients with multiple uncontrolled conditions suggest that clinicians may be appropriately identifying and managing high-risk patients with uncontrolled hypertension, those patients with higher CVD risk who are more likely to benefit from BP control.

Our results are concordant with other studies indicating better quality of care for medically complex patients [13,14]. Heisler *et al*. also reported higher medication intensification rates among patients with higher BPs [15]. We found that other uncontrolled CVD risk factors had a similar effect and did not in fact 'compete' for the clinician's attention to BP management. Because many of these patients had additional medical conditions besides diabetes and/or hyperlipidemia, further investigation is needed to assess the effect of conditions unrelated to CVD risk on decision-making and disease management [16]. Particularly, it is not clear how painful arthritis, for example, influences CVD risk factor management.

These findings reinforce our and others' work suggesting that inaction by clinicians is not necessarily clinical inertia [11,17]. Inaction may include both appropriate and inappropriate clinical care. Appropriate inaction, such as not intensifying medications in the setting of non-adherence, reflects the challenge of applying guidelines to the care of individual patients balanced with meeting population-based quality indicators. We noted a considerable amount of appropriate action in the situation of medication intensification for the highest risk patients (BP at least 10 mmHg above goal and uncontrolled diabetes and hypertension).

On the other hand, intensification rates observed when the BP was closer to goal were quite low and may represent appropriate inaction. In these modest ranges of elevation, clinicians may be more cautious, possibly weighing the modest benefits against the potential harms of a more intense regimen. The difference in management of patients close to BP goals and those with higher BP highlights the intricacy of providing guideline-concordant yet patient-centered care in primary care. Additionally, BP measurement is known to be imprecise, and low elevations may require close monitoring to assess the stability of the level. Further, the risks of polypharmacy are real and an important potentially appropriate reason to forego intensification of the BP regimen. In addition, a single BP level does not reflect past efforts, which may have resulted in considerable BP lowering and cardiovascular risk reduction. Unfortunately, dichotomous quality indicators, including the one assessing BP control, do not acknowledge the subtleties involved in 'real world' clinical decision making, nor do they account for appropriate inaction. In fact, tying rewards to a simplistic quality indicator that overlooks potentially clinically appropriate inaction may result in unintended consequences, creating incentives for polypharmacy and raising costs.

We observed that clinicians were possibly inappropriately overestimating the benefit derived from controlled diabetes and/or hyperlipidemia. BP medication intensification rates were consistently the lowest for individuals with either controlled diabetes or hyperlipidemia even with systolic BP > 10 mm Hg above goal. These rates were lower than for individuals with only uncontrolled hypertension, and including the number of hypertension, diabetes, and hyperlipidemia medications in the model did not change this effect. Controlled diabetes and/or hyperlipidemia lowers but does not eliminate the risk of CVD, so efforts to improve BP control remain important in reducing CVD risk [18]. In light of recent data from the ACCORD study, control of systolic BP to 120 mm Hg failed to demonstrate a decrease in major cardiovascular events in patients with diabetes compared to less stringent BP (systolic BP < 140 mm Hg) [19,20]. Regardless, lack of treatment intensification when BP is notably above goal among patients with known but controlled diabetes and/or hyperlipidemia could be the target of quality improvement activities, and may represent true clinical inertia.

Our study of clinicians who practice in a healthcare system with strong quality improvement initiatives and incentives to control BP allowed us to observe whether clinicians implement guidelines differently based on individual patient factors, including the benefits and risks of medication intensification. These individual patient factors and complex clinical decisions may not be apparent by measuring quality indicators using administrative data and may only be documented with observational data.

Our study has several limitations. Although the sample of 13 clinicians at one VA facility caring for mostly male patients raises issues of generalizability, our results confirm past reports of better quality of care with more medically complex patients. With only 13 participating clinicians, we were not able to explore clinician characteristics that could be contributing to variations in care, and this may warrant further investigation with a larger study. Additionally, we may have seen higher rates of medication intensification than typical practice settings because clinicians knew we were asking them to document whether they changed medications. Our sample was too small to confirm our preliminary findings suggesting that BP medication intensification patterns were similar for controlled versus uncontrolled hyperlipidemia and for controlled versus uncontrolled diabetes. We were also unable to conduct multivariate analyses of these findings, which differ from past reports indicating that hyperlipidemia is more often ignored compared to uncontrolled diabetes or hypertension [21,22]. These findings require confirmation in larger studies. Important patient-level factors that could contribute to suboptimal BP control and clinical inaction, such as cost or adherence issues, were not available.

## Summary

The presence of uncontrolled diabetes and hyperlipidemia increased the likelihood of BP medication intensification in patients with uncontrolled hypertension. This effect was observed regardless of the level of BP elevation, but the magnitude of the effect was greater if BP was further from goal. These findings suggest that clinicians correctly identified and treated the highest risk hypertensive patients likely to receive the greatest benefit with BP medication intensification. Lower BP medication intensification rates seen in patients with modest BP elevations and in patients with controlled diabetes and/or hyperlipidemia may represent appropriate clinical inaction and inappropriate clinical inertia respectively, but further investigation is needed. Our study reflects the complex decision making process involved in providing medical care that balances clinical guideline-concordance (based on the benefit to groups or populations) and patient-centeredness to patients with multiple uncontrolled conditions. The implementation of quality indicators that measure medication intensification, account for the clinical risks and benefits of medication intensification, and assess both appropriate clinical actions and inactions are needed to maximize high quality, patient-centered care.

## Competing interests

The authors declare that they have no competing interests.

## Authors' contributions

AS, EF, JA, JH, TK, DL, and MS contributed to the conception and design of the study. EF, BA, ML, and MS acquired and analyzed the data. AS, EF, JH, DL, and MS drafted the manuscript. All authors contributed to data interpretation, critical revisions of the manuscript, and read and approved the final manuscript.
